# The Genome-Wide Identification of the GST Gene Family and Functional Characterization of *PsGSTF8* in Anthocyanin Accumulation in Chinese Plum Fruit (*Prunus salicina*)

**DOI:** 10.3390/biology15131002

**Published:** 2026-06-25

**Authors:** Yuan Wang, Menghan Wu, Siyu Li, Longji Li, Yanke Geng, Gaopu Zhu, Danfeng Bai, Shaobin Yang, Fangdong Li, Taishan Li, Minggui Gong, Gaigai Du

**Affiliations:** 1 College of Food and Bioengineering, Henan University of Science and Technology, Luoyang 471023, China; xiaoyuan.sun@foxmail.com; 2 Research Institute of Non-Timber Forestry, Chinese Academy of Forestry, Zhengzhou 450003, China; wumenghanyd@163.com (M.W.); lxlisiyu@163.com (S.L.); 12456agdf@gmail.com (L.L.); yankegeng987@163.com (Y.G.); zhugaopu@163.com (G.Z.); baidanfeng1993@163.com (D.B.); ysb1966327@aliyun.com (S.Y.); lifangdong66@163.com (F.L.); litaishan@caf.ac.cn (T.L.)

**Keywords:** Chinese plum, GSTs, GST gene family, anthocyanins, fruit coloration, *PsGSTF8*

## Abstract

Anthocyanins are major determinants of both the color and nutritional quality of fruit. Their accumulation in plant tissues depends on biosynthesis as well as efficient transport into the vacuole, a process in which glutathione S-transferases (GSTs) are believed to play an important role. Through genome-wide identification and analysis, this study identified 39 Chinese plum (*Prunus salicina*) glutathione S-transferase (*PsGST*) genes. Phylogenetic analysis grouped these *PsGST* genes into seven subfamilies. Dispersed duplication appeared to be the main driver of family expansion, and the duplicated genes appear to have largely evolved under purifying selection. The cultivar ‘Fengweimeigui’ (FWMG), with purple peel and red pulp, and the cultivar ‘Fengweihuanghou’ (FWHH), with yellow peel and yellow pulp, were used as contrasting materials because of their distinct anthocyanin pigmentation. We then profiled anthocyanin accumulation and the expression patterns of all *PsGST* genes across fruit development using quantitative real-time PCR (qRT-PCR). *PsGSTF8* was identified as a candidate gene, and its expression patterns in both peel and pulp were consistent with anthocyanin accumulation, with higher relative transcript levels in the deeply colored cultivar ‘FWMG’ and undetectable expression in ‘FWHH’. The transient overexpression of *PsGSTF8* in Chinese plum fruit promoted anthocyanin accumulation, and the complementation of the *Arabidopsis thaliana* transparent testa 19 (*tt19*) mutant further supported the potential role of *PsGSTF8* in anthocyanin accumulation and GST-mediated transport. These results provide new clues for understanding potential GST-mediated anthocyanin transport and accumulation and offer a basis for the further investigation of the molecular mechanisms underlying fruit coloration in Chinese plum.

## Simple Summary

Chinese plum is an important fruit crop with a wide range of fruit colors, among which red and purple cultivars are particularly appreciated by consumers. This coloration is mainly attributed to the accumulation of anthocyanins. In addition to biosynthesis, the efficient transport of these pigments into the vacuole is required for stable anthocyanin accumulation in fruit. Glutathione S-transferases (GSTs) are believed to mediate this transport process; however, the specific GST members involved in anthocyanin accumulation in Chinese plum remain unclear. We identified and characterized 39 *PsGST* genes in the Chinese plum genome. Fruit color phenotypes, anthocyanin content and gene expression profiles were jointly analyzed to screen candidate *PsGST* genes potentially associated with anthocyanin accumulation. Among these, *PsGSTF8* was selected for functional validation, and its role in anthocyanin accumulation was supported by functional assays. In summary, these findings provide new insights into GST-mediated anthocyanin transport in Chinese plum and offer a valuable candidate gene for molecular breeding aimed at improving fruit coloration and related quality traits.

## 1. Introduction

Anthocyanins are a key group of secondary metabolites of plants, which apart from fruit coloration also provide antioxidant protection and stress resistance [1,2]. Their stable accumulation not only is regulated by biosynthesis but also requires efficient transport from the cytosol into the vacuole [3]. To date, three major mechanisms have been proposed for anthocyanin transport. Of these, glutathione S-transferase (GST)-mediated transport is considered an important step in anthocyanin transport into the vacuole [4].

GSTs are members of a large, evolutionarily conserved family of enzymes that are widely distributed in eukaryotes and prokaryotes [5]. However, plant genomes generally contain larger numbers of GST genes than many other organisms, mainly due to the expansion of two subfamilies, the GSTU and plant-specific GSTF subfamilies [6]. Most GST genes linked to anthocyanin accumulation and transport are members of the GSTF subfamily, with only a few belonging to the GSTU subfamily [7]. Some GSTs are thought to associate with anthocyanins in the cytosol, thereby helping to maintain their stability and facilitate their transport into the vacuole [8].

Genome-wide analyses have identified and characterized GST family members and revealed their structural and evolutionary features in diverse plant species. Early evidence for the involvement of GSTs in anthocyanin accumulation came from maize, where *Bz2* was shown to be required for anthocyanin accumulation and vacuolar deposition [9]. This idea gained further support when researchers observed that *Arabidopsis thaliana* mutants lacking *AtGSTF12* (also known as *TT19*) fail to accumulate anthocyanins properly [10]. Since then, evidence has accumulated that GST-mediated anthocyanin transport may represent a relatively conserved mechanism in plants. For example, in cyclamen, *CkmGST3* can recover pigment accumulation in the *Arabidopsis* transparent testa 19 (*tt19*) background, implying a function in anthocyanin accumulation [11].

In fruit crops, GSTs have also been increasingly recognized as important contributors to anthocyanin accumulation and fruit coloration. In grapevine, *VviGST1*, *VviGST3*, and *VviGST4* exhibit functional specialization and show distinct preferences for different flavonoids, with *VviGST3* favoring proanthocyanidin transport and *VviGST4* contributing to anthocyanin and proanthocyanidin deposition [12]. In kiwifruit, the *AcGST1* expression pattern is closely associated with anthocyanin accumulation [13], and in blueberry, *VcGSTF8* has been identified as a strong candidate gene associated with fruit anthocyanin accumulation [14]. In Rosaceae fruit species, GST-mediated anthocyanin transport has also been linked to fruit pigmentation. For example, in peach, *PpGST1* appears to be mainly involved in anthocyanin transport rather than proanthocyanidin transport [15], while in apple, *MdGSTU12* shows a strong correlation with anthocyanin accumulation in the fruit peel and has been functionally associated with anthocyanin transport [16]. Additional studies of *RAP* in strawberry, *PcGSTF12* in pear, and *PavGST1* in sweet cherry also support the view that GSTs may play a conserved role in flavonoid accumulation, particularly anthocyanin-related pigmentation, within this family [17,18,19].

Recent studies have improved our understanding of how anthocyanins accumulate and contribute to fruit coloration in Chinese plum. Red pulp or red peel cultivars generally accumulate higher levels of anthocyanins, whereas yellow or lightly colored genotypes tend to accumulate lower levels. Cyanidin-3-O-glucoside represented the dominant anthocyanin compound detected in Chinese plum fruit [20]. The transcriptional regulation of anthocyanin accumulation is partly mediated by *PsMYB10* family members. Among them, *PsMYB10.1* is closely related to peel coloration, while *PsMYB10.2* is associated with red pulp genotypes and promotes pulp pigmentation through the activation of downstream genes, including *PsUFGT* [21]. In ‘Kongxin’ plum, the red pigmentation of the peel is linked to the enhanced transcription of key structural genes in the anthocyanin biosynthetic pathway, represented by *PsPAL1*, *PsC4H*, *PsCHS1*, and *PsDFR2* [22]. Light and temperature treatments also affect anthocyanin accumulation; in ‘Akihime’, suitable conditions enhance peel anthocyanin accumulation through a *PsMYB10.1*-mediated pathway [23]. During the low-temperature storage of ‘Xiushi’ plum, *PsERF1B* participates in cold-triggered pigmentation by forming a regulatory module with *PsMYB10.1* and *PsbHLH3*, activating *PsUFGT* transcription and enhancing anthocyanin biosynthesis [24].

Overall, previous studies in Chinese plum have mainly focused on MYB-mediated transcriptional regulation and anthocyanin biosynthetic structural genes, whereas less attention has been paid to the post-biosynthetic intracellular transport and vacuolar accumulation of anthocyanins. Several Chinese plum *(Prunus salicina*) glutathione S-transferase (*PsGST*) genes have been suggested to function downstream of *PsMYB10.1* or *PsMYB10.2* and may be involved in anthocyanin transport. However, information on the genome-wide characteristics of the *PsGST* family, their expression patterns in cultivars with contrasting pigmentation, and the potential functions of individual *PsGST* genes remains limited [25]. Therefore, the further characterization of *PsGST* genes may help improve our understanding of anthocyanin accumulation in Chinese plum fruit.

In the present study, we performed a comprehensive genome-wide analysis of the GST gene family in Chinese plum. ‘Fengweimeigui’ (FWMG; purple peel and red pulp) and ‘Fengweihuanghou’ (FWHH; yellow peel and yellow pulp) were selected because they represent distinct fruit pigmentation phenotypes. This clear phenotypic contrast provides suitable material for exploring GST genes potentially associated with anthocyanin transport and accumulation in Chinese plum fruit. To investigate the potential roles of *PsGST* genes in Chinese plum pigmentation, we combined expression analysis, transient expression assays, and genetic complementation in the *Arabidopsis* transparent testa 19 (*tt19*) mutant to screen candidate *PsGST* genes and assess the potential role of *PsGSTF8* in anthocyanin accumulation and GST-mediated transport. These findings may provide useful information for further understanding the post-biosynthetic regulation of anthocyanin accumulation and fruit pigmentation in Chinese plum.

## 2. Materials and Methods

### 2.1. Plant Materials

Fruits of two Chinese plum cultivars, ‘Fengweimeigui’ (FWMG) and ‘Fengweihuanghou’ (FWHH), were harvested from the Prunoideae Germplasm Resource Nursery at the Yuanyang Experimental Base of the Chinese Academy of Forestry. For each cultivar, three trees of similar age and showing normal growth without visible disease or pest symptoms were selected as independent biological replicates. Fruit materials were collected at three developmental stages, namely the green stage (G), color-break stage (B), and mature stage (M). These stages were defined based on days after full bloom (DAFB) and visual fruit coloration characteristics. Specifically, the G, B, and M stages corresponded to 90, 120, and 150 DAFB, respectively, in both cultivars (Table S1). The G stage was characterized by predominantly green peel, the B stage by the initial appearance of cultivar-specific peel coloration, and the M stage by fully developed cultivar-specific peel coloration. For each cultivar at each developmental stage, fruits were collected from three individual trees, with 10 fruits from each tree pooled as one biological replicate, yielding three biological replicates per cultivar at each stage (*n* = 3) and a total of 30 fruits per cultivar per stage. After collection, the fruit peel and pulp were rapidly separated, snap-frozen in liquid nitrogen, and stored at −80 °C.

The *Arabidopsis thaliana* materials used in this study included the wild-type Columbia-0 ecotype (Col-0) and the *tt19* mutant (SALK_113164C). Seeds of the *tt19* mutant were obtained from the AraShare Arabidopsis Genetic Resource Center (http://www.arashare.cn/). All Arabidopsis plants were grown in a controlled growth chamber under a 16 h light/8 h dark photoperiod at 22 ± 2 °C, with a light intensity of 100 μmol m^−2^ s^−1^.

### 2.2. Identification and Physicochemical Characterization of GST Gene Family Members

The genome sequences, gene annotation files (GFF3), and predicted protein sequences of Chinese plum were acquired from the GDR (https://www.rosaceae.org/; accessed on 15 October 2025). For comparative purposes, corresponding genomic datasets of apricot (*Prunus armeniaca*), sweet cherry (*Prunus avium*), peach (*Prunus persica*), and Japanese apricot (*Prunus mume*) were likewise retrieved from the same database [26]. *Arabidopsis thaliana* GST protein sequences, obtained from TAIR (https://www.arabidopsis.org; accessed on 15 October 2025), served as queries for a BLASTP (https://blast.ncbi.nlm.nih.gov/Blast.cgi?PAGE=Proteins, accessed on 15 October 2025) homology search against the Chinese plum predicted protein database under an E-value cutoff of 1 × 10^−5^ [27]. As a complementary approach, the HMM profiles of the conserved GST_N and GST_C domains were sourced from the InterPro (Pfam) database (https://www.ebi.ac.uk/interpro; accessed on 20 October 2025) and screened against the predicted proteins with HMMER v3.4 (http://hmmer.org; accessed on 27 October 2025) at the same E-value threshold [28]. After merging candidate sequences obtained from BLASTP and HMMER, redundant or truncated sequences were removed. Domain architecture was then confirmed via the SMART database (https://smart.embl.de/; accessed on 25 October 2025) [29]. The finalized sequences are listed in Table S2. The key physicochemical parameters including the protein length, isoelectric point (pI), molecular weight (MW), instability index and grand average of hydropathicity (GRAVY) were estimated using ProtParam on the ExPASy platform (https://web.expasy.org/protparam/; accessed on 29 October 2025) (Table S3).

### 2.3. Sequence Alignment and Phylogenetic Tree Construction

GST protein sequences from *Arabidopsis thaliana*, Chinese plum, apricot, sweet cherry, peach, and Japanese apricot were aligned using MAFFT v7.526 [30]. The alignment was subsequently manually checked and trimmed to remove poorly aligned, gap-rich, and non-conserved regions. Based on this refined alignment, a maximum likelihood (ML) phylogenetic tree was inferred in MEGA v11.0.13 with branch support evaluated over 1000 bootstrap replicates [31,32]. The resulting tree was plotted and labeled using the package ggtree (v4.0.5) from Bioconductor in R v4.5.1 [33].

### 2.4. Gene Structure, Conserved Motif, and Conserved Domain Analysis of PsGSTs

The structural organization of *PsGST* genes was analyzed using the Chinese plum genome annotation data. Genomic and CDS sequences for each identified GST member were extracted with TBtools v1.120 and used to draw exon–intron organization diagrams. The conserved domain positions were annotated in NCBI Conserved Domain Database and displayed together with TBtools to visualize gene structures and domain architecture. Motif analysis was performed with MEME v5.5.8, setting the maximum number of motifs to 15.

### 2.5. Chromosomal Localization, Gene Duplication, Comparative Synteny, and Ka/Ks Analysis

The genomic positions of *PsGST* members were displayed on Chinese plum chromosomes using TBtools. Duplication types among *PsGST* family members were classified with the duplicate_gene_classifier module of MCScanX v1.0, based on gene collinearity and physical positions [34]. Interspecific synteny across the five *Prunus* species was analyzed using the JCVI module in TBtools, and cross-species syntenic homologous gene pairs of *PsGSTs* were identified. For each duplicated gene pair, coding sequence alignments were generated with ParaAT v2.0. For each duplicated gene pair, Ka and Ks were estimated using the Nei–Gojobori (NG) model in KaKs_Calculator. Selection patterns after duplication were inferred from the corresponding Ka/Ks values [35].

### 2.6. Analysis of Transcription Factor Binding Sites and Cis-Regulatory Elements

The promoter analysis of *PsGST* genes began with the selection of a representative transcript for each gene from the Chinese plum genome annotation file. The 2000 bp sequence upstream of each transcription start site was retrieved and defined as the putative promoter region. The promoter sequences were submitted to PlantCARE (https://bioinformatics.psb.ugent.be/webtools/plantcare/html/; accessed on 28 October 2025) for the prediction and functional classification of putative cis-regulatory elements (CREs) [36]. The transcription factor binding sites (TFBSs) were predicted using the FIMO tool in MEME Suite v5.5.3, with a significance threshold of *p* ≤ 1 × 10^−5^. The resulting datasets from both analyses were then combined and visualized using with the R package ggplot2.

### 2.7. Anthocyanin Content Determination

Total anthocyanin levels were measured based on absorbance differences under two pH conditions using the pH differential method [37]. In brief, 0.5 g of fresh tissue was homogenized after liquid nitrogen freezing and then extracted with 1 mL of 1% HCl–methanol solution at 4 °C for 24 h without light exposure. The extract was clarified by centrifugation at 12,000 rpm for 10 min, after which the supernatant was retained for subsequent analysis. The extraction solution was diluted with KCl buffer (0.025 mol·L^−1^) at pH 1.0 and sodium acetate buffer (0.4 mol·L^−1^) at pH 4.5. Absorbance at 520 nm and 700 nm was measured after room temperature equilibration. The absorbance difference was derived from A = [(A_520_ − A_700_) pH 1.0 − (A_520_ − A_700_) pH 4.5]. Total anthocyanins were quantified as cyanidin-3-glucoside equivalent content and reported as mg per 100 g fresh weight [38].

### 2.8. qRT-PCR Analysis

We extracted total RNA using TRIzol reagent (TransGen Biotech, Beijing, China). Reverse transcription was performed using the PrimeScript™ RT reagent kit to generate cDNA (TaKaRa, Dalian, China). A LightCycler^®^ 480 II system (Roche, Basel, Switzerland) was used for qRT-PCR with SYBR Green qPCR Premix (2×) (Koton Biotechnology, Beijing, China). Each sample included three independent biological replicates. Transcript abundance was estimated using the 2^−ΔΔCt^ method, with *PsActin* serving as the internal reference gene [39]. Supplementary Table S4 lists all primer sequences, which were generated with Primer Premier 5.0 and ordered from Sangon Bioengineering Co., Ltd. (Shanghai, China).

### 2.9. Construction of Overexpression Vector

The complete coding sequence of *PsGSTF8* was amplified from cDNA synthesized from the peel of mature ‘FWMG’ fruit. The pGreenII 62-SK plasmid was linearized by double digestion with SpeI and EcoRI. Primers with homologous extensions matching the vector ends were designed in Primer Premier 5.0 (Table S5). After gel recovery and the purification of the PCR products, the *PsGSTF8* fragment was inserted into the pGreenII 62-SK vector using the ClonExpress II One Step Cloning Kit (Vazyme, Nanjing, China), resulting in the construction of the CaMV 35S promoter-driven *PsGSTF8* overexpression vector 35S::*PsGSTF8*. Following validation by sequencing, the recombinant vector was introduced into *Agrobacterium tumefaciens* GV3101.

### 2.10. Transient Overexpression of PsGSTF8

To ensure the reproducibility of *Agrobacterium*-mediated injection infiltration and subsequent sampling, preliminary infiltration tests were first performed using different Chinese plum cultivars. Based on these tests, mature ‘WeiDi’ (WD) fruit was selected as the most suitable material for *PsGSTF8* transient overexpression. ‘WD’ was selected only for transient functional validation because its fruits had relatively uniform maturity, no visible mechanical damage, and a firm but injectable texture. In contrast, the soft tissue of ‘FWMG’ fruit tended to cause the leakage of the bacterial suspension, local tissue collapse, and inconsistent sampling after injection, whereas the excessively firm tissue of ‘FWHH’ fruit made needle penetration difficult, increased resistance during syringe plunger depression, caused the local cracking of the dense pulp tissue during injection, and restricted the uniform diffusion of the infiltration suspension. Therefore, ‘WD’ fruit was used to improve the consistency of injection infiltration, *PsGSTF8* transient expression, anthocyanin accumulation analysis, and downstream sampling. The fruits were harvested as described in Section 2.1.

*Agrobacterium cultures* harboring either the 35S::*PsGSTF8* (*PsGSTF8*-OE) construct or the empty vector were resuspended in infiltration buffer according to Zhao et al. [15]. For each fruit, the empty vector and *PsGSTF8*-OE suspensions were injected into two opposite sides of the same fruit using a sterile 1 mL syringe, with the needle inserted approximately 3–4 mm into the fruit tissue. A total of 2 mL of *Agrobacterium* suspension was injected into each fruit, with 1 mL of each suspension applied to the corresponding injection site. The fruits were kept at 25 °C for 5 d after injection. Three biological replicates were used, each consisting of infiltrated tissues from 10 independently injected fruits. For each replicate, tissues from the empty vector- and *PsGSTF8*-OE-infiltrated sides were collected separately and pooled by treatment. The pooled samples were immediately frozen in liquid nitrogen and stored at −80 °C until further analysis. The collected infiltrated tissues were used for *PsGSTF8* expression analysis and total anthocyanin content determination using the pH differential method, a spectrophotometric method described in Section 2.7.

### 2.11. Generation of Arabidopsis tt19 Lines Overexpressing PsGSTF8

Positive *Agrobacterium* colonies were isolated and cultured. Bacterial cells were resuspended in infiltration medium to an OD_600_ of 0.8–1.0. *Arabidopsis tt19* mutant plants were grown in a controlled growth under the same controlled conditions as described in Section 2.1. The plants were transformed using the floral dip method [40]. Harvested T1 seeds were sown on 1/2 MS medium containing 30 mg/L kanamycin for selection and cultured under the same conditions described above. The selected transgenic lines were propagated to the T3 generation. T3 seeds from independent transgenic lines were germinated and grown under the same controlled conditions, and the resulting seedlings were used for phenotypic observation, a qRT-PCR analysis of *PsGSTF8* expression and anthocyanin content determination. The method for anthocyanin determination is described in Section 2.7, and primer information is provided in Table S5.

### 2.12. Statistical Analysis

Data were analyzed by GraphPad Prism v10.6.0 and presented as the mean ± standard deviation (SD). Group differences were assessed by a two-tailed Student’s *t*-test (two groups) or one-way ANOVA with Tukey’s multiple comparisons test (more than two groups).

## 3. Results

### 3.1. Identification and Physicochemical Characterization of GST Gene Family in Chinese Plum

Based on the genome and proteome information of Chinese plum, the candidate GST sequences were first retrieved by HMM-based domain search and then validated for their domain architectures. This systematic screening workflow resulted in the identification of 39 high-confidence GST gene family members. The PsGST proteins ranged in length from 86 to 421 amino acids, with corresponding molecular weights (MWs) ranging from 9.23 to 47.92 kDa. The pI values ranged from 4.24 to 9.80, with 23 proteins classified as acidic proteins (pI < 7) and 16 as basic proteins (pI > 7). The instability index varied from 25.00 to 56.31. Using 40 as the threshold, 16 proteins were predicted to be unstable, whereas the remaining 23 were predicted to be stable. In addition, 36 PsGST proteins had negative GRAVY values, indicating that most members were predicted to be hydrophilic (Table S6).

### 3.2. Phylogenetic Relationships of GST Proteins from Different Species

**Figure 1:**
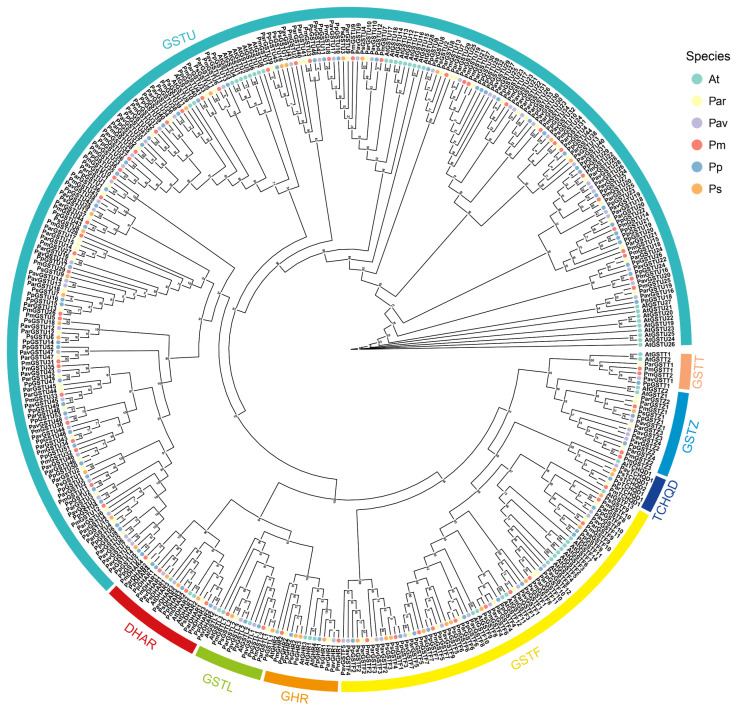
A maximum likelihood (ML) phylogenetic tree of GST proteins from *P. salicina* (Ps), *P. armeniaca* (Par), *P. avium* (Pav), *P. persica* (Pp), *P. mume* (Pm), and *A. thaliana* (At), constructed based on aligned GST protein sequences. Arabidopsis GST proteins were included as references for subfamily classification. The terminal labels indicate GST protein names from different species, and the colored dots next to the labels represent species identity. Colored arcs in the outer ring indicate different GST subfamilies, including GSTU, GSTF, GSTT, GSTZ, TCHQD, GHR, GSTL, and DHAR. Numbers at the nodes indicate bootstrap support values.

A dataset comprising 326 GST proteins from Chinese plum, apricot, sweet cherry, peach, and Japanese apricot was assembled for evolutionary relationship analysis. These sequences were combined with 56 GST proteins from *Arabidopsis thaliana* as references. The aligned GST protein dataset was used for ML phylogenetic analysis (Figure 1). According to the tree topology and the classical classification system of the *Arabidopsis* GST family, all GST proteins were assigned to their corresponding subfamilies. The *Arabidopsis* GST proteins served as subfamily references, and the classification of PsGSTs was supported by their clustering with *Arabidopsis* members from the corresponding GST subfamilies.

In total, 382 GST proteins, including 39 PsGSTs, 71 ParGSTs, 72 PavGSTs, 68 PmGSTs, 76 PpGSTs, and 56 AtGSTs, were classified into eight subfamilies (Table S6). Among the identified subfamilies, GSTU contained the highest number of members, totaling 239, whereas GSTF was the second largest, with 67 members. Most *PsGST* members were grouped within the GSTU and GSTF subfamilies, consistent with the expansion pattern commonly observed in plant GST families. It should be noted that no GSTT subfamily members were identified within the PsGST family. Accordingly, the Chinese plum GST family was assigned to seven subfamilies, including Tau (GSTU), Phi (GSTF), Lambda (GSTL), DHAR, Zeta (GSTZ), TCHQD, and GHR (Table S2). Among these, DHAR, GSTL, and GHR clustered within the same major branch, suggesting a relatively close phylogenetic relationship. Based on the phylogenetic tree, PsGST members did not form large Chinese plum-specific clades. Instead, most PsGSTs were grouped with GST proteins from other *Prunus* species and Arabidopsis GSTs from the corresponding subfamilies. This pattern was particularly evident in the GSTU and GSTF subfamilies.

### 3.3. Analysis of Gene Structures, Conserved Motifs, and Conserved Domains of PsGSTs

**Figure 2:**
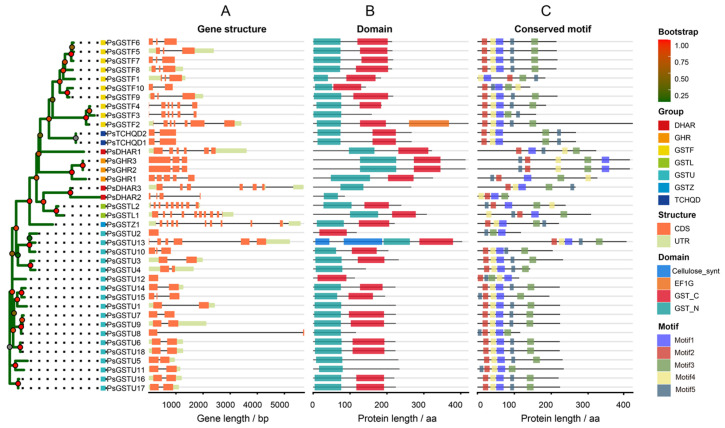
Phylogenetic relationships, gene structures, conserved domains, and conserved motifs of *PsGST* family: (**A**) Exon–intron organization of *PsGST* genes. Gene models are displayed with orange boxes for coding regions, light green boxes for non-coding regions, and gray lines for introns. (**B**) Conserved domain architecture of PsGST proteins. Different colors of boxes represent different conserved domains, including GST_N, GST_C, EF1G, and Cellulose_synt. (**C**) Conserved motif patterns across PsGST proteins are displayed. Motifs are displayed as colored boxes (1–5), and scale bar represents protein’s length.

Comparative gene structure analysis showed clear differences in exon–intron organization across subfamilies of the *PsGST* family, while members within the same subfamily generally exhibited more conserved gene structural patterns. Within the GSTU subfamily, most members contained two exons and one intron (13/18). Gene structures in the GSTF subfamily were relatively diverse compared with those in other GST subfamilies. *PsGSTF1*, *PsGSTF5*, *PsGSTF6*, *PsGSTF7*, *PsGSTF8*, and *PsGSTF9* each contained three exons and two introns; *PsGSTF3* and *PsGSTF4* contained six exons and five introns; *PsGSTF2* contained seven exons and six introns; and *PsGSTF10* contained only two exons and one intron. The GSTZ subfamily contained 11 exons and 10 introns (Figure 2A).

The majority of PsGST proteins were predicted to contain the canonical GST_N and GST_C domains (26/39), whereas some members showed variation in domain composition. In particular, ten proteins were predicted to contain only the GST_N domain, and two proteins were predicted to contain only the GST_C domain. Based on the current genome annotation and domain prediction results, these sequences were retained as PsGST candidates, although they may represent partial or truncated predictions. In addition, EF1G and Cellulose_synt domains were additionally predicted in PsGSTF2 and PsGSTU13, respectively (Figure 2B; Table S7). In total, five conserved motifs ranging from 11 to 21 amino acids were identified in the PsGST proteins (Figure S1). Most PsGST members had the full complement of five motifs, except for the absence of Motif 5 in PsDHAR2 and the presence of only three motifs (1, 3, and 5) in PsGSTU2 (Figure 2C).

### 3.4. Chromosomal Localization, Gene Duplication, and Synteny Analysis of PsGST Genes

**Figure 3:**
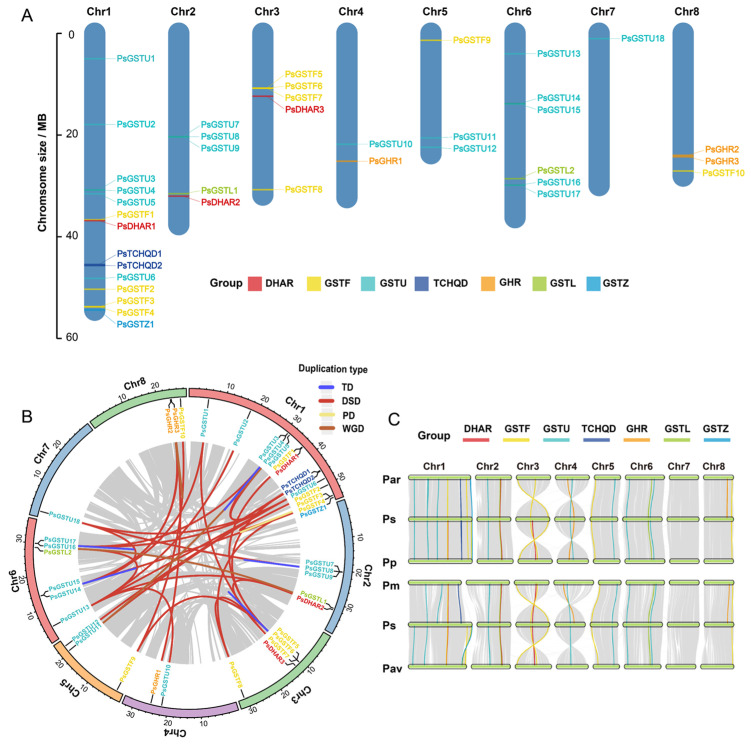
Genomic distribution, duplication modes, and syntenic relationships of *PsGST* genes: (**A**) Chromosomal distribution of *PsGST* genes in Chinese plum. Different colors indicate different GST subfamilies. (**B**) Duplication patterns of *PsGST* genes in Chinese plum genome. Eight chromosomes are displayed in outer circle, gray links represent intragenomic syntenic blocks and colored links indicate different duplication types, including dispersed duplication (DSD), tandem duplication (TD), whole-genome duplication (WGD), and proximal duplication (PD). (**C**) Comparative synteny between Chinese plum and other *Prunus* species. Green blocks represent chromosomes of each species, gray links represent genome-wide collinear regions, and colored links highlight collinear GST gene pairs from different subfamilies.

Chromosomal mapping revealed that the 39 *PsGST* genes were unevenly distributed across the eight chromosomes of Chinese plum (Figure 3A). Among them, chromosome 1 harbored the largest number of *PsGST* members (14 genes), followed by chromosome 6 with six members, while chromosomes 2 and 3 each carried five members. Three *PsGST* genes were located on each of chromosomes 5 and 8, two on chromosome 4, and only one on chromosome 7. In addition, adjacent gene clusters consisting of *PsGSTU7*–*PsGSTU9* and *PsGSTF5*–*PsGSTF7* were identified on chromosomes 2 and 3, respectively.

An analysis of the duplication patterns within the *PsGST* gene family identified 46 duplicated pairs, including 35 dispersed duplication (DSD) pairs, six tandem duplication (TD) pairs, three whole-genome duplication (WGD) pairs, and two proximal duplication (PD) pairs (Figure 3B). DSD represented the dominant duplication type among the duplicated gene pairs (76.1%), indicating that it may have been a major contributor to PsGST family expansion. Comparative collinearity analysis between Chinese plum and the other four *Prunus* species identified 21, 23, 31, and 19 collinear homologous GST gene pairs with apricot, peach, Japanese apricot, and sweet cherry, respectively (Figure 3C).

### 3.5. Selection Pressure Analysis of Duplicated PsGST Gene Pairs

**Figure 4:**
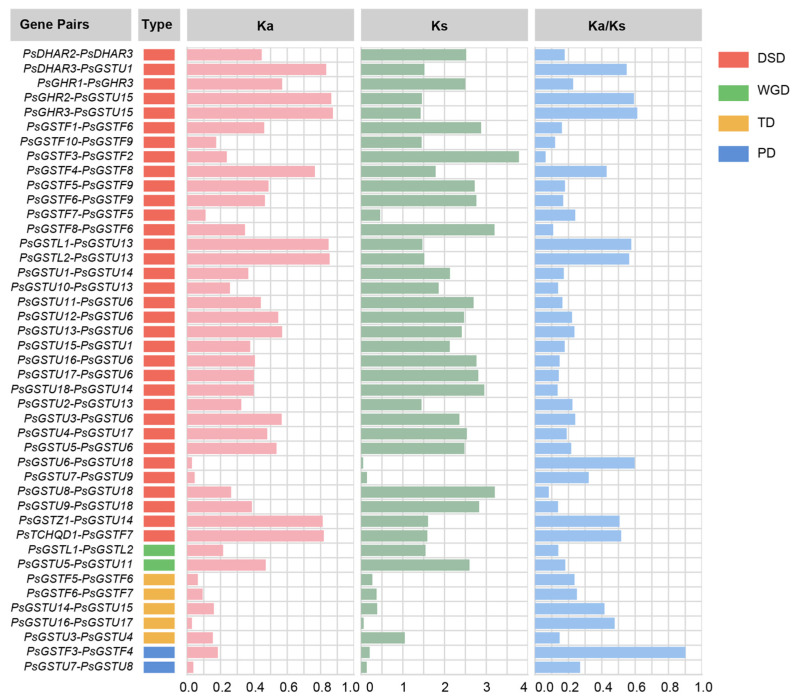
The Ka, Ks, and Ka/Ks values of duplicated *PsGST* gene pairs. The leftmost column lists the duplicated *PsGST* gene pairs, and the ‘Type’ column indicates the corresponding duplication mode. Horizontal bars show the calculated Ka values, Ks values, and Ka/Ks ratios for each duplicated gene pair, with pink, green, and blue bars indicating Ka, Ks, and Ka/Ks, respectively. Different colors in the ‘Type’ column represent four duplication modes: dispersed duplication (DSD, red), whole-genome duplication (WGD, green), tandem duplication (TD, yellow), and proximal duplication (PD, blue).

To evaluate whether duplicated *PsGST* gene pairs experienced selective pressure after duplication, Ka, Ks, and Ka/Ks values were calculated using the Nei–Gojobori (NG) model. Selection patterns were then inferred from these metrics (Figure 4). In total, 44 duplicated *PsGST* gene pairs were retained after discarding gene pairs with a Ka or Ks value equal to 0. The mean Ka/Ks ratio was 0.287 (0.062–0.901), with most values between 0.108 and 0.269. Overall, the duplicated *PsGST* genes in Chinese plum mainly evolved under purifying selection.

### 3.6. Transcription Factor Binding Site Prediction and Cis-Regulatory Element Analysis of PsGST Genes

**Figure 5:**
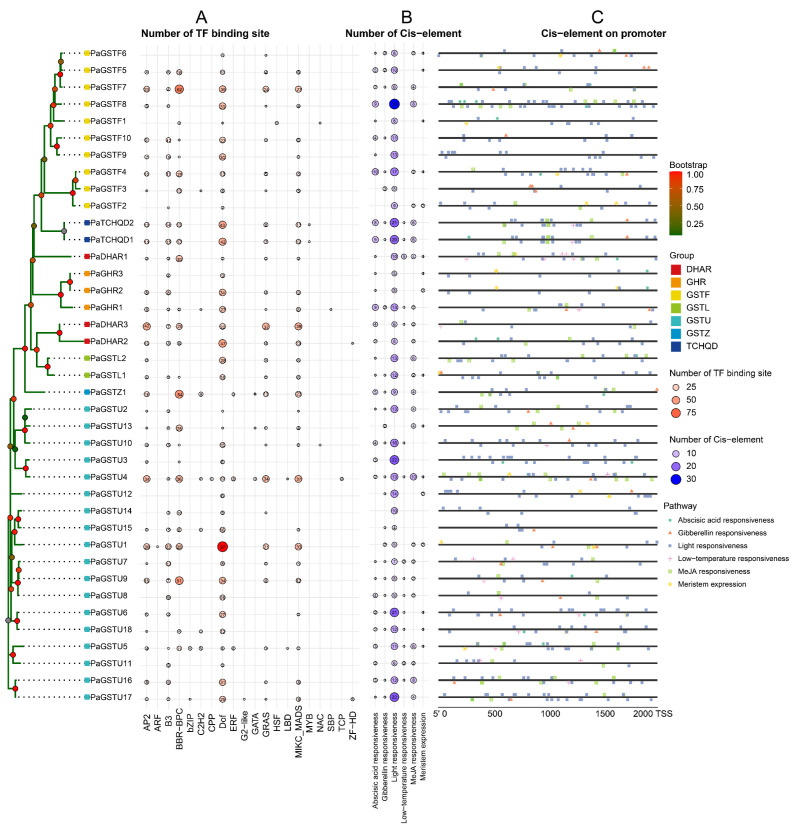
Predicted transcription factor binding sites (TFBSs) and cis-regulatory elements (CREs) in the promoter regions of *PsGST* genes: (**A**) The left panel displays the phylogenetic tree of PsGST proteins, with node colors from green to red indicating increasing bootstrap support and colored blocks beside gene names marking subfamily affiliation. The right panel shows a bubble plot of the number of predicted TFBSs across different transcription factor families in each *PsGST* promoter. (**B**) The profiles of predicted major CREs in *PsGST* promoters. Rows represent *PsGST* promoters, columns represent CRE types, and bubble size and color intensity indicate the abundance of each CRE type. (**C**) The spatial distribution of predicted CREs within the 2000 bp upstream promoter regions of *PsGST* genes. Different colors and shapes denote distinct functional categories of CREs.

The potential regulatory features of *PsGST* promoters were explored by analyzing the 2000 bp upstream regions of *PsGST* genes for TFBSs and CREs (Figure 5). The *PsGST* promoters were predicted to contain various TFBSs, predominantly Dof (743), BBR-BPC (443), MIKC_MADS (306), AP2 (282), and B3 (198) (Figure 5A). Among these, Dof-binding sites were the most prevalent in *PsGST* promoters, although their numbers varied markedly among genes. The highest number was observed in the *PsGSTU1* promoter, which contained 97 Dof-binding sites, whereas no Dof-binding site was predicted in the *PsGSTZ1* promoter.

The functional classification of predicted CREs showed that *PsGST* promoters contained elements associated with abscisic acid responsiveness, gibberellin responsiveness, low-temperature responsiveness, light responsiveness, methyl jasmonate responsiveness, and meristem expression (Figure 5B). Among the CREs identified in *PsGST* promoters, light-responsive elements constituted the predominant category, accounting for 61.2% of all identified CREs. They were followed by abscisic acid-responsive elements (14.7%) and methyl jasmonate-responsive elements (14.1%). These predicted promoter features suggest that some *PsGST* genes may be responsive to light-related regulation.

### 3.7. Phenotypic Characteristics of Peel and Pulp Coloration and Total Anthocyanin Content at Different Stages of Fruit Development

**Figure 6:**
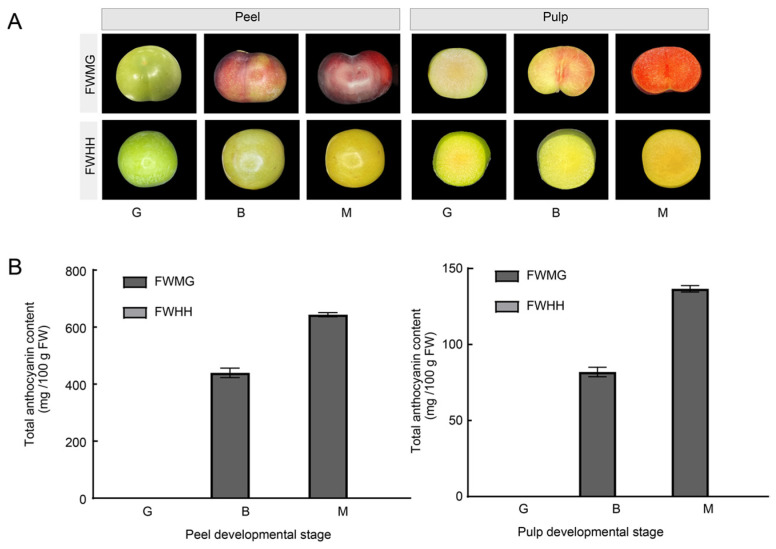
Peel and pulp coloration and total monomeric anthocyanin content in ‘FWMG’ and ‘FWHH’ fruits at different developmental stages: (**A**) Representative phenotypes of the peel and pulp of ‘FWMG’ and ‘FWHH’ fruits at the green (G), color-break (B), and mature (M) stages. (**B**) Total monomeric anthocyanin content in the peel and pulp of ‘FWMG’ and ‘FWHH’ fruits at the G, B, and M stages, determined using the pH differential method and expressed as mg/100 g fresh weight (FW). The left and right bar charts show the anthocyanin content in peel and pulp, respectively. The absence of a ‘FWHH’ bar indicates that total monomeric anthocyanin was not detected in ‘FWHH’ in the corresponding tissue/stage. Data are presented as the mean ± SD of three biological replicates, and error bars indicate SD. ‘FWMG’, ‘Fengweimeigui’; ‘FWHH’, ‘Fengweihuanghou’.

During fruit development, ‘FWMG’ gradually developed purple and red coloration in both the peel and pulp, reaching the highest color intensity at the M stage. In contrast, the peel of ‘FWHH’ remained predominantly green to yellow during development, and no red pigmentation was observed in the pulp (Figure 6A). Consistent with the phenotypic observations, total anthocyanin content in both the peel and pulp of ‘FWMG’ fruits increased as development progressed, reaching the highest level at the M stage, with greater accumulation detected in the peel than in the pulp. By comparison, total anthocyanin content was not detected in either the peel or pulp of ‘FWHH’ fruit samples at the G, B, or M stages under the conditions used in this study (Figure 6B).

### 3.8. Spatiotemporal Expression Patterns of GST Family Genes in Two Chinese Plum Cultivars

**Figure 7:**
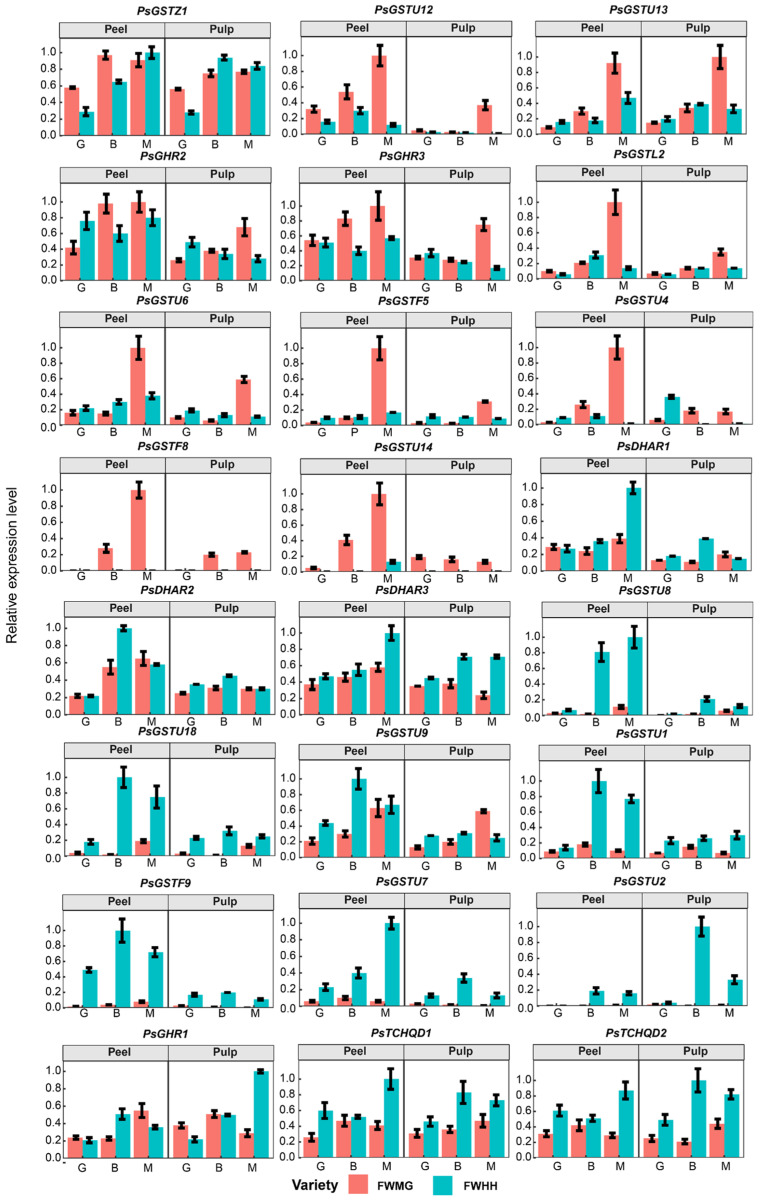
The qRT-PCR analysis of selected *PsGST* genes in ‘Fengweimeigui’ (FWMG) and ‘Fengweihuanghou’ (FWHH) fruit peel and pulp across developmental stages. Each panel depicts the expression pattern of an individual gene in peel and pulp tissues. G, B, and M represent the green, color-break, and mature stages of fruit development, respectively, as defined in this study. Red and cyan bars denote ‘FWMG’ and ‘FWHH’, respectively. Expression values were normalized to the maximum value detected among all tested samples for each gene; therefore, the data indicate relative expression patterns within each gene rather than absolute expression levels among different genes. Data represent the mean ± SD of three independent biological replicates, with the remaining *PsGST* gene expression data shown in Figure S2.

The transcript abundance of the 39 *PsGST* members was assessed in ‘FWMG’ and ‘FWHH’ peel and pulp tissues across fruit development using qRT-PCR (Figure 7 and Figure S2). Because the expression values in the heatmap were normalized to the maximum value for each gene, these data were used to compare expression patterns within each gene across samples, rather than absolute transcript levels among different genes. Except for *PsGSTU3*, *PsGSTU11*, and *PsGSTU15*, whose expression was not detected under the conditions examined in this study, the remaining 36 genes were classified into three groups with distinct expression profiles (Figure S3). Among these, Group I genes tended to show higher relative expression at the B and M stages in ‘FWMG’ peel and at the M stage in pulp (Figure 7). Notably, *PsGSTF8* showed high relative expression in the peel and pulp of the ‘FWMG’ fruit at both the B and M stages, whereas its expression was not detected in the corresponding stages of ‘FWHH’ fruit. Pearson correlation analysis was performed between *PsGST* gene expression and total anthocyanin content across 12 matched samples (Figure S4). Among the analyzed *PsGST* genes, *PsGSTF8* showed the strongest positive correlation with anthocyanin content (r = 0.96, *p* < 0.001). Several other genes, including *PsGSTL2*, *PsGSTU4*, *PsGSTU12*, *PsGSTU14*, *PsGSTF5*, *PsGSTU6*, *PsGHR2*, and *PsGHR3*, also showed positive correlations with anthocyanin content. However, their correlation coefficients were lower than the coefficient for *PsGSTF8*. Together, the expression pattern, anthocyanin accumulation profile, and correlation analysis support *PsGSTF8* as the most suitable candidate for further functional validation.

Genes in Group II exhibited distinct tissue and stage specificity but lacked a consistent expression pattern (Figures S2 and S3). Most Group III genes showed relatively higher expression in ‘FWHH’ peel and pulp at the B and M stages, within their respective expression profiles, implying a potential negative correlation with anthocyanin accumulation (Figure 7 and Figure S3). Consistently, Pearson correlation analysis showed that several Group III genes were negatively correlated with anthocyanin content, with *PsGSTF2* and *PsGSTF6* showing significant negative correlations (*p* < 0.05; Figure S4).

### 3.9. Transient Overexpression of PsGSTF8 in ‘WD’ Fruit

**Figure 8:**
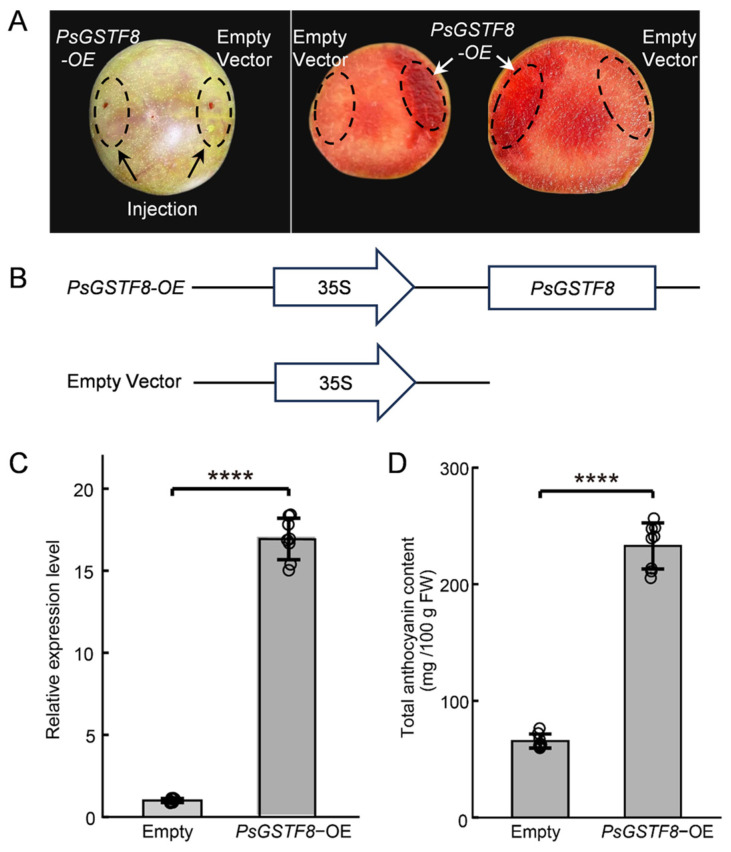
The effect of transient *PsGSTF8* overexpression on anthocyanin accumulation in Chinese plum ‘WeiDi’ (WD) fruit: (**A**) Representative phenotypes of ‘WD’ fruit tissues at 5 d after Agrobacterium-mediated infiltration with the empty vector or the *PsGSTF8* overexpression construct (*PsGSTF8*-OE). Dashed circles indicate the infiltrated regions, and arrows indicate the injection sites. (**B**) Schematic diagrams of the CaMV 35S-driven *PsGSTF8* overexpression construct and the empty vector. (**C**) Relative *PsGSTF8* expression in tissues infiltrated with the empty vector or *PsGSTF8*-OE, as determined by qRT-PCR. (**D**) Total monomeric anthocyanin content in tissues infiltrated with the empty vector or *PsGSTF8*-OE, determined using the pH differential method and expressed as mg/100 g fresh weight (FW). Data are presented as the mean ± SD of three biological replicates, each consisting of pooled infiltrated tissues from 10 independently injected fruits. Statistical significance was assessed using a two-tailed Student’s *t*-test. Asterisks indicate significant differences between the *PsGSTF8*-OE and empty vector groups (****, *p* < 0.0001).

To evaluate whether *PsGSTF8* could promote anthocyanin accumulation in fruit, we constructed a *PsGSTF8* overexpression vector and used an empty vector as a control. ‘WD’ fruits with uniform size and physiological status were selected as transient overexpression materials. Agrobacterium-mediated infiltration was performed by injecting Agrobacterium suspensions containing the empty vector or the *PsGSTF8*-OE recombinant plasmid into two opposite positions on the same fruit. The fruits were incubated at 25 °C for 5 d after infiltration. Phenotypic observation showed that the tissue transiently overexpressing *PsGSTF8* developed darker coloration than the empty vector control (Figure 8A,B). The transient overexpression of *PsGSTF8* in ‘WD’ fruits was validated by qRT-PCR, which showed an approximately 16-fold-higher expression level relative to the empty vector control (*p* < 0.0001; Figure 8C). To determine whether the visible pigmentation was associated with a measurable change in anthocyanin content, total monomeric anthocyanins in the infiltrated tissues were quantified using the pH differential method (a spectrophotometric method). *PsGSTF8*-overexpressing tissues accumulated 247.6 mg/100 g FW total monomeric anthocyanins, representing a 3.54-fold increase compared with the empty vector treatment (*p* < 0.0001; Figure 8D). These results suggest that *PsGSTF8* may promote anthocyanin accumulation in plum fruit tissue under the transient overexpression conditions used in this study.

### 3.10. Functional Analysis of PsGSTF8 in tt19 Mutant

**Figure 9:**
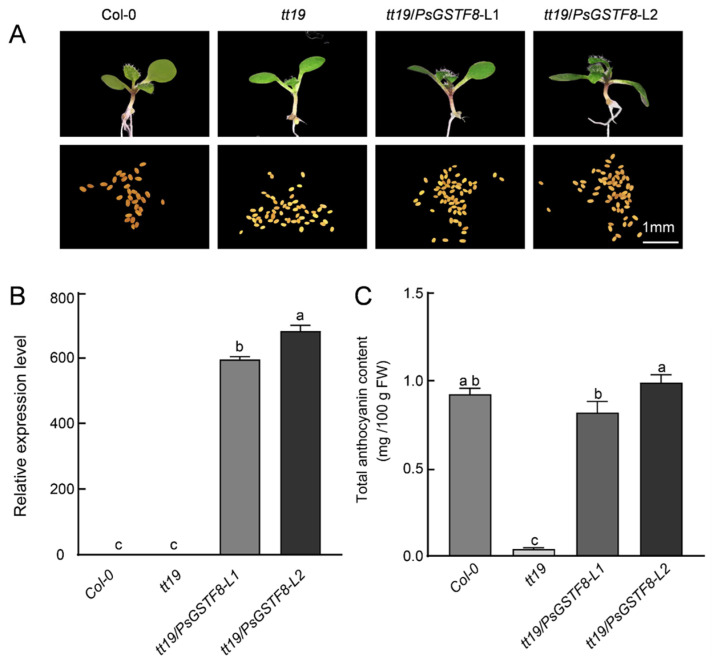
The functional complementation of the *Arabidopsis tt19* mutant by *PsGSTF8*: (**A**) A phenotypic comparison of wild-type Col-0, the *tt19* mutant, and two 35S::*PsGSTF8* transgenic complementation lines (L1 and L2), including hypocotyl pigmentation and mature seed coat color. Scale bar = 1 mm. (**B**) Relative *PsGSTF8* expression in Col-0, *tt19*, and the transgenic lines L1 and L2, as determined by qRT-PCR, with *AtActin2* used as the internal reference gene. (**C**) Total monomeric anthocyanin content in Col-0, the *tt19* mutant, and the two *PsGSTF8* complementation lines grown on 1/2 MS medium containing 5% sucrose. Data are presented as the mean ± SD of three biological replicates. Different letters denote significant differences (one-way ANOVA with Tukey’s multiple comparisons test, *p* < 0.05).

To evaluate the potential role of *PsGSTF8* in anthocyanin accumulation and GST-mediated transport, 35S::*PsGSTF8* was expressed in the *Arabidopsis tt19* mutant for heterologous functional complementation [10]. Under the growth conditions used in this study, the hypocotyls of wild-type Col-0 seedlings were red. The *tt19* mutant, which is defective in an anthocyanin-related GST gene, showed reduced red pigmentation in the hypocotyls. In contrast, the *PsGSTF8* transgenic lines restored the red hypocotyl phenotype, while the seed coat color of mature seeds was partially restored toward the Col-0 phenotype (Figure 9A). qRT-PCR analysis confirmed *PsGSTF8* expression in the transgenic lines L1 and L2, whereas *PsGSTF8* transcripts were not detected in Col-0 or *tt19* plants (Figure 9B). Total monomeric anthocyanin content, quantified using the pH differential method, was significantly increased in L1 and L2 compared with the *tt19* mutant, levels approximately 20-fold higher than that in *tt19*. The anthocyanin levels in L1 and L2 were comparable to those in Col-0 and were significantly higher than those in *tt19* (*p* < 0.05; Figure 9C). These results suggest that *PsGSTF8* expression was associated with increased anthocyanin accumulation in the *tt19* mutant background.

## 4. Discussion

The stable accumulation of anthocyanins in plant vacuoles is a major determinant of red, purple, and blue coloration in fruits [41]. GSTs are conserved multifunctional proteins involved in the intracellular transport and vacuolar accumulation of anthocyanins [42]. In the present study, 39 GST members were identified in Chinese plum, providing a basis for exploring GST-mediated anthocyanin transport in this species. Phylogenetic analysis classified these members into seven subfamilies, whereas no GSTT members were identified in the current Chinese plum genome annotation. This pattern may be associated with lineage-specific gene loss, annotation limitations, or differential retention during evolution. Compared with the GST families reported in some closely related Rosaceae species, the relatively small size of the *PsGST* family suggests that GST expansion in Chinese plum may have been limited [43]. Four duplication types were identified in the *PsGST* gene family, with DSD accounting for the highest proportion, whereas TD events were relatively rare. This pattern differs from previous reports in some Rosaceae species, where GST family expansion was mainly associated with TD [43], suggesting that the mechanisms underlying GST family expansion may vary among Rosaceae fruit trees. The relatively low frequency of TD events may have contributed to the limited expansion of the *PsGST* family. These results suggest that Chinese plum maintains a relatively conserved GST repertoire, in which specific GSTF and GSTU members may play roles in flavonoid transport, oxidative stress responses, environmental adaptation, and anthocyanin-related fruit coloration [44].

Predicted promoter CREs provide important clues for understanding the potential regulatory mechanisms of *PsGST* genes. The high abundance of predicted light-responsive elements in *PsGST* promoters is consistent with previous reports in other fruit crops, suggesting that some *PsGST* genes may be associated with light-related regulation during anthocyanin accumulation and fruit color development. For example, UV-B and white light treatments induced the expression of *MiGSTF8*, *MiGSTF9*, and *MiGSTU7* and were accompanied by anthocyanin accumulation in fruit peel [45], while in litchi, bag removal enhanced light exposure and promoted *LcGST4* expression in association with anthocyanin accumulation [46]. The presence of elements related to abscisic acid response, gibberellin response, methyl jasmonate response, low-temperature response, and meristem expression further suggests that *PsGST* genes may be regulated by multiple hormonal, developmental, and environmental signals. However, these predictions do not directly demonstrate actual responsiveness to these signals, and their functional significance requires further validation through light or hormone treatment experiments, promoter activity assays, and transcription factor binding experiments.

Classical GST genes such as maize *BZ2*, petunia *AN9*, and *Arabidopsis TT19* have been shown to mediate anthocyanin transport, and the *tt19* mutant is widely used for functional validation [9,10,42]. Similar roles have also been reported for GST genes in several fruit crops. For instance, *LcGST4* in litchi, *MdGSTF6* in apple, *PpGST1* in peach, *MrGST1* in Chinese bayberry, *PavGST1* in sweet cherry, and *PcGST57* in pear have been reported to participate in anthocyanin transport or fruit coloration [15,19,46,47,48,49]. Their expression is frequently controlled by MYB transcription factors, suggesting that anthocyanin-related GST genes may act as downstream functional components of the anthocyanin regulatory network. The divergent expression patterns of the *PsGST* genes in this study suggest the potential for functional specialization in the GST family. The expression profile of *PsGSTF8* was particularly notable, with the transcript levels of this gene paralleling anthocyanin accumulation in ‘FWMG’, while no such trend was observed in the pale-colored cultivar ‘FWHH’. This expression divergence was consistent with the anthocyanin concentration and fruit color of the two cultivars, and this is a major reason why *PsGSTF8* was selected as a promising candidate for investigating its role in anthocyanin accumulation.

Because anthocyanin-related GSTs are generally not considered to directly catalyze anthocyanin biosynthesis, they are believed to contribute to the accumulation of anthocyanins in the vacuole by binding anthocyanins and facilitating their transport into the vacuole. *PsGSTF8* may contribute to anthocyanin transport and vacuolar accumulation in Chinese plum. Given that the association between *PsGSTF8* expression and anthocyanin accumulation was mainly observed in two contrasting cultivars, this result should not be interpreted as evidence that *PsGSTF8* is a universal determinant of Chinese plum fruit color without further validation in additional cultivars or broader germplasm resources. Beyond *PsGSTF8*, several other *PsGST* genes showed expression patterns that partially paralleled anthocyanin accumulation in ‘FWMG’. However, similar associations were not observed in ‘FWHH’. This suggests that the relationship between *PsGST* expression and anthocyanin accumulation may depend on genotype, tissue type, developmental stage, and the cultivar-specific anthocyanin biosynthetic background. Functional differentiation among GSTs has been reported previously. For instance, grapevine *VviGST1*, *VviGST3*, and *VviGST4* display distinct substrate preferences for anthocyanins and proanthocyanidins, and in *Medicago*, *MtrGSTF7* regulates flavonoid accumulation in an organ- and stage-specific manner [12,50]. In this context, the high expression level of some Group III genes in the low-anthocyanin material ‘FWHH’ may reflect their involvement in other metabolic processes, antioxidant regulation, or fruit development, rather than directly promoting anthocyanin accumulation.

The transient overexpression of *PsGSTF8* in ‘WD’ fruit deepened fruit coloration and significantly increased anthocyanin content, supporting a potential role for *PsGSTF8* in fruit anthocyanin accumulation. Similar roles have been reported for anthocyanin-associated GST genes, including kiwifruit *AcGST1*, peach *PpGST1*, sweet cherry *PavGST1*, and pear *PcGST57*, suggesting partial functional conservation among anthocyanin-related GST genes in fruit crops [13,15,19,49]. However, because the transient assay was conducted in the non-target cultivar ‘WD’, the results should be interpreted with the consideration of cultivar-specific physiological and genetic backgrounds. Differences in endogenous anthocyanin levels, fruit developmental status, hormone balance, the basal expression of anthocyanin biosynthetic genes, and upstream regulatory activity between ‘WD’ and the main experimental cultivars ‘FWMG’ and ‘FWHH’ may influence the strength of the observed phenotype. Thus, the transient assay in ‘WD’ provides important evidence that *PsGSTF8* has the capacity to promote anthocyanin accumulation under transient overexpression conditions, but further validation in the target cultivars, together with stable transformation or gene-editing approaches, is needed to further clarify its role in Chinese plum fruit coloration. The overexpression of *PsGSTF8* restored the anthocyanin-deficient hypocotyl phenotype of the *Arabidopsis tt19* mutant, suggesting that *PsGSTF8* may be involved in anthocyanin accumulation and GST-mediated transport. This is consistent with reports that peach *PpGST1* and apple *MdGSTF6* can complement the *tt19* anthocyanin-deficient phenotype [15,47]. However, seed coat coloration was only partially restored, suggesting that *PsGSTF8* may have limited capacity to complement the proanthocyanidin-deficient phenotype of *tt19* or that its function may depend on substrate type, tissue context, or heterologous genetic background. Whether *PsGSTF8* is also associated with proanthocyanidin accumulation or transport requires further investigation.

In summary, the expression patterns and functional assays support *PsGSTF8* as a promising candidate gene involved in anthocyanin accumulation in Chinese plum fruit. The comparative evolutionary and promoter analyses further suggest that the *PsGST* family may be relatively stable but functionally differentiated and that some members may be regulated by light, hormonal, low-temperature, and developmental signals that may influence fruit coloration or stress responses. Nevertheless, the results obtained from transient assays in the non-target cultivar should be interpreted cautiously. Further studies, such as subcellular localization, protein–anthocyanin binding assays, stable transformation, promoter activity assays, and an analysis of upstream transcriptional regulation, are needed to clarify how *PsGSTF8* contributes to fruit coloration in Chinese plum.

## 5. Conclusions

In this study, a genome-wide analysis of the GST gene family in Chinese plum characterized the evolutionary, structural, and regulatory features of *PsGST* members, offering a basis for the further investigation of GST-mediated anthocyanin transport and accumulation in this species. Expression profiling and functional assays suggested that *PsGSTF8* may be a promising candidate associated with anthocyanin accumulation and potentially involved in GST-mediated transport. These findings improve our understanding of anthocyanin-related fruit coloration in Chinese plum. After further validation in diverse germplasm resources and breeding populations, genetic variation associated with *PsGSTF8* may provide useful information for developing molecular markers associated with fruit color and anthocyanin accumulation. Such information could support future marker-assisted selection for fruit color improvement and anthocyanin-related quality traits. Therefore, *PsGSTF8* may serve as a promising candidate gene for further functional analysis and potential breeding applications in Chinese plum.

## Data Availability

The original contributions presented in this study are included in the article/Supplementary Materials. Further inquiries can be directed to the corresponding authors.

## Supplementary Materials

The following supporting information can be downloaded at https://www.mdpi.com/article/10.3390/biology15131002/s1, Figure S1. Sequence logo analysis of conserved motifs in PsGST proteins; Figure S2. Relative expression levels of *PsGST* family genes determined by qRT-PCR in peel and pulp of ‘FWMG’ and ‘FWHH’ fruits at developmental stages; Figure S3. Hierarchical clustering analysis of *PsGST* family genes based on qRT-PCR expression data; Figure S4. Pearson correlation analysis between *PsGST* gene expression and total anthocyanin content across 12 matched samples. Table S1. Fruit developmental stages and sampling dates used in this study; Table S2. GST protein sequences identified in *Arabidopsis thaliana*, *Prunus armeniaca*, *Prunus avium*, *Prunus mume*, *Prunus persica*, and *Prunus salicina*; Table S3. Protein physicochemical properties; Table S4. Primers used for qRT-PCR; Table S5. Sequences related to *PsGSTF8* expression vector construction; Table S6. The number of GSTs of six species in each subclass; Table S7. Genomic locations, gene structure features, and conserved domain annotations of GST family genes in *Prunus salicina*.
