# Supplementary figures and images for: The Genome-Wide Identification of the GST Gene Family and Functional Characterization of PsGSTF8 in Anthocyanin Accumulation in Chinese Plum Fruit (Prunus salicina)
Source: Biology (Basel). 2026 Jun 25;15(13):1002. doi: 10.3390/biology15131002 (PMC13359690; doi:10.3390/biology15131002)

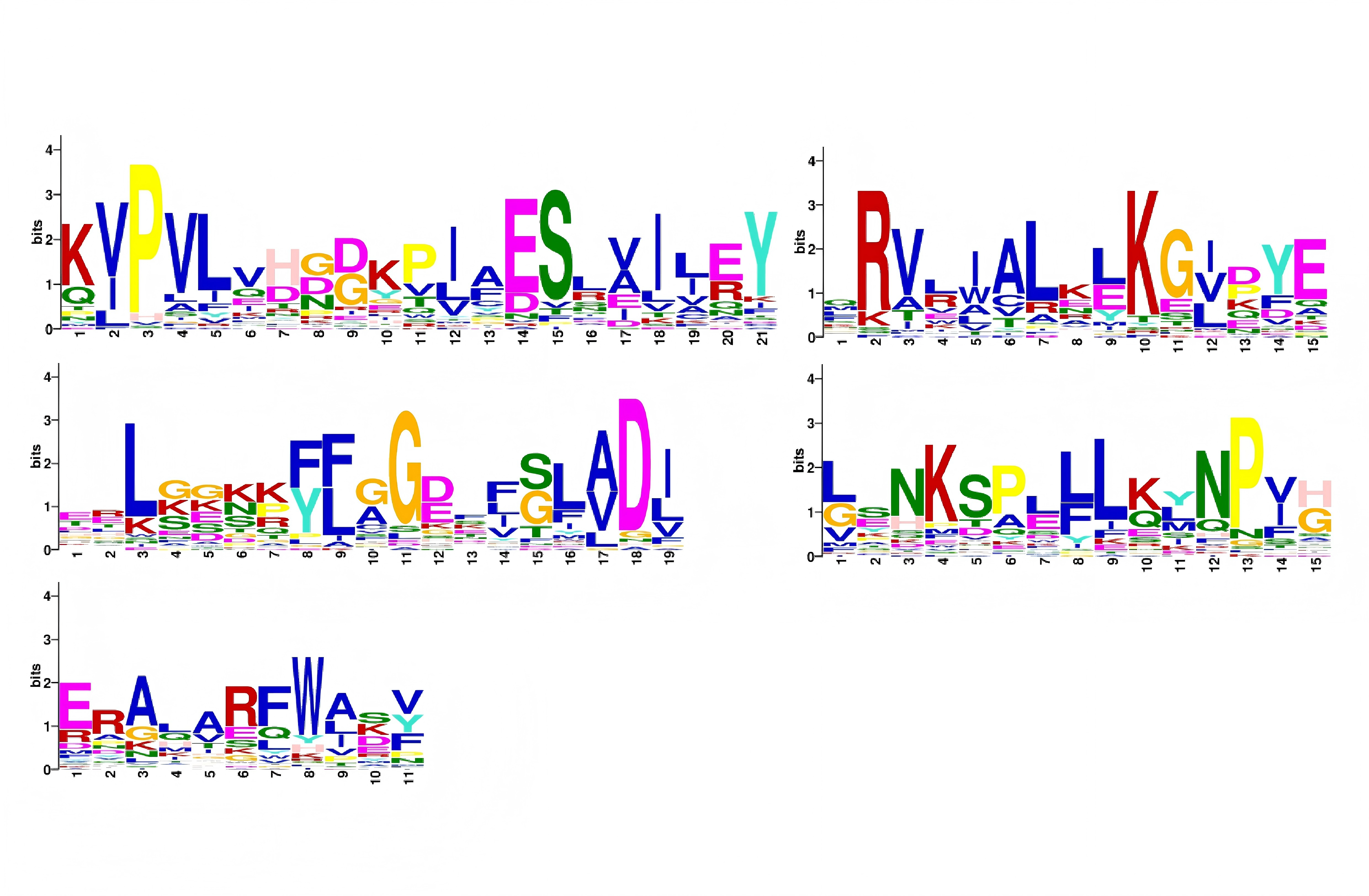

Supplement: Supplementary file 1 [file biology-15-01002-s001.zip › supplementary Figure S1.png]

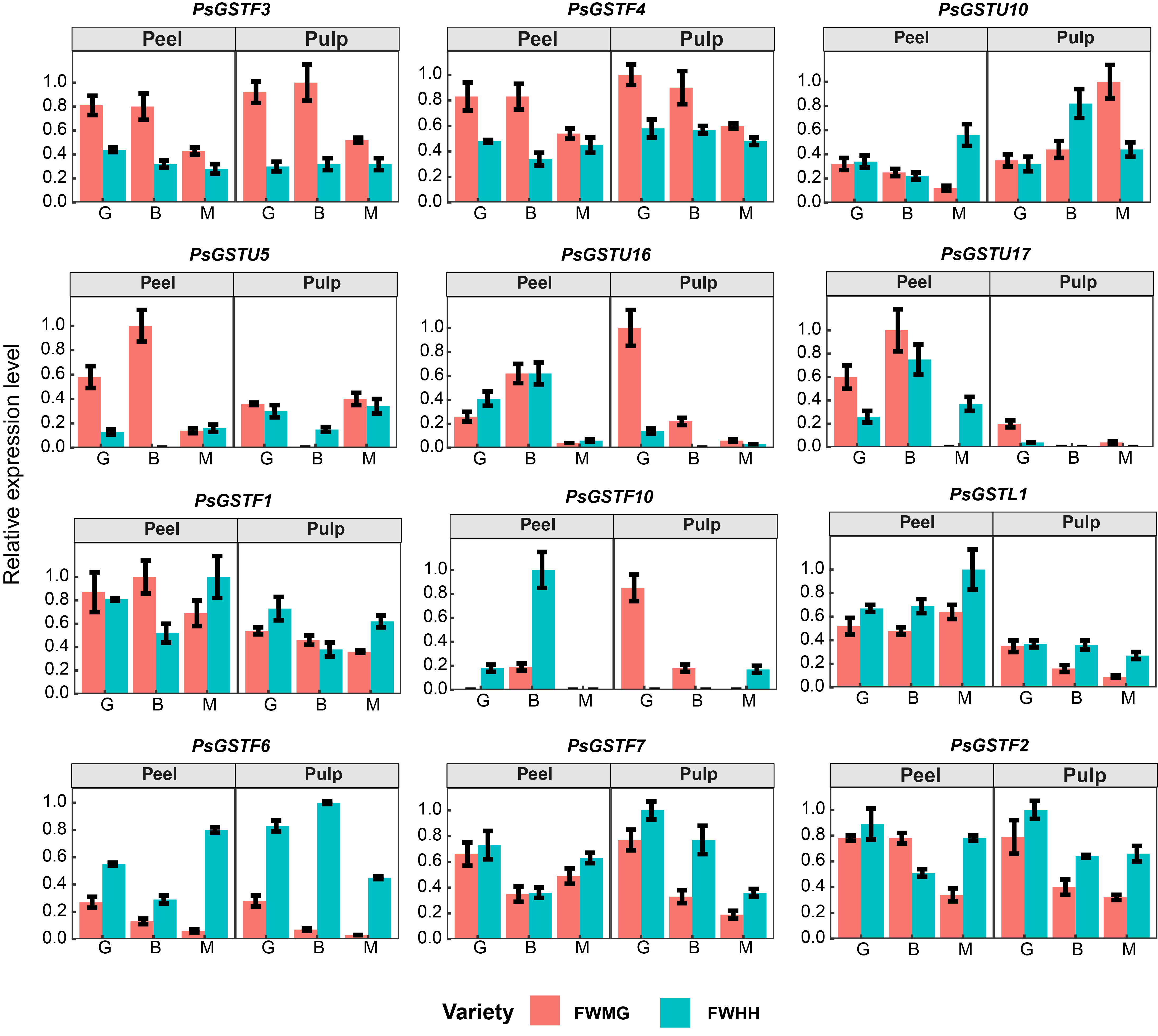

Supplement: Supplementary file 1 [file biology-15-01002-s001.zip › supplementary Figure S2.png]

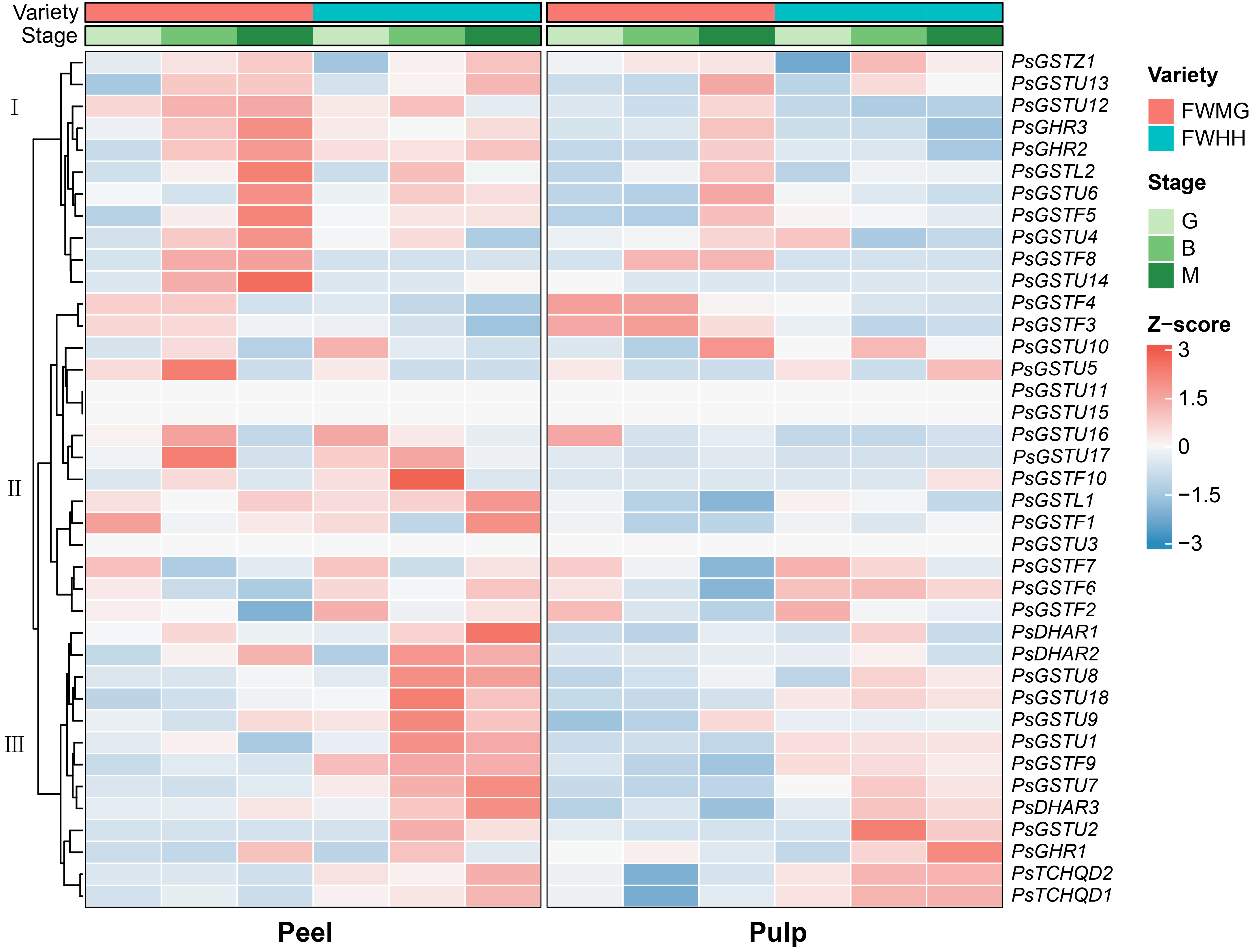

Supplement: Supplementary file 1 [file biology-15-01002-s001.zip › supplementary Figure S3.png]

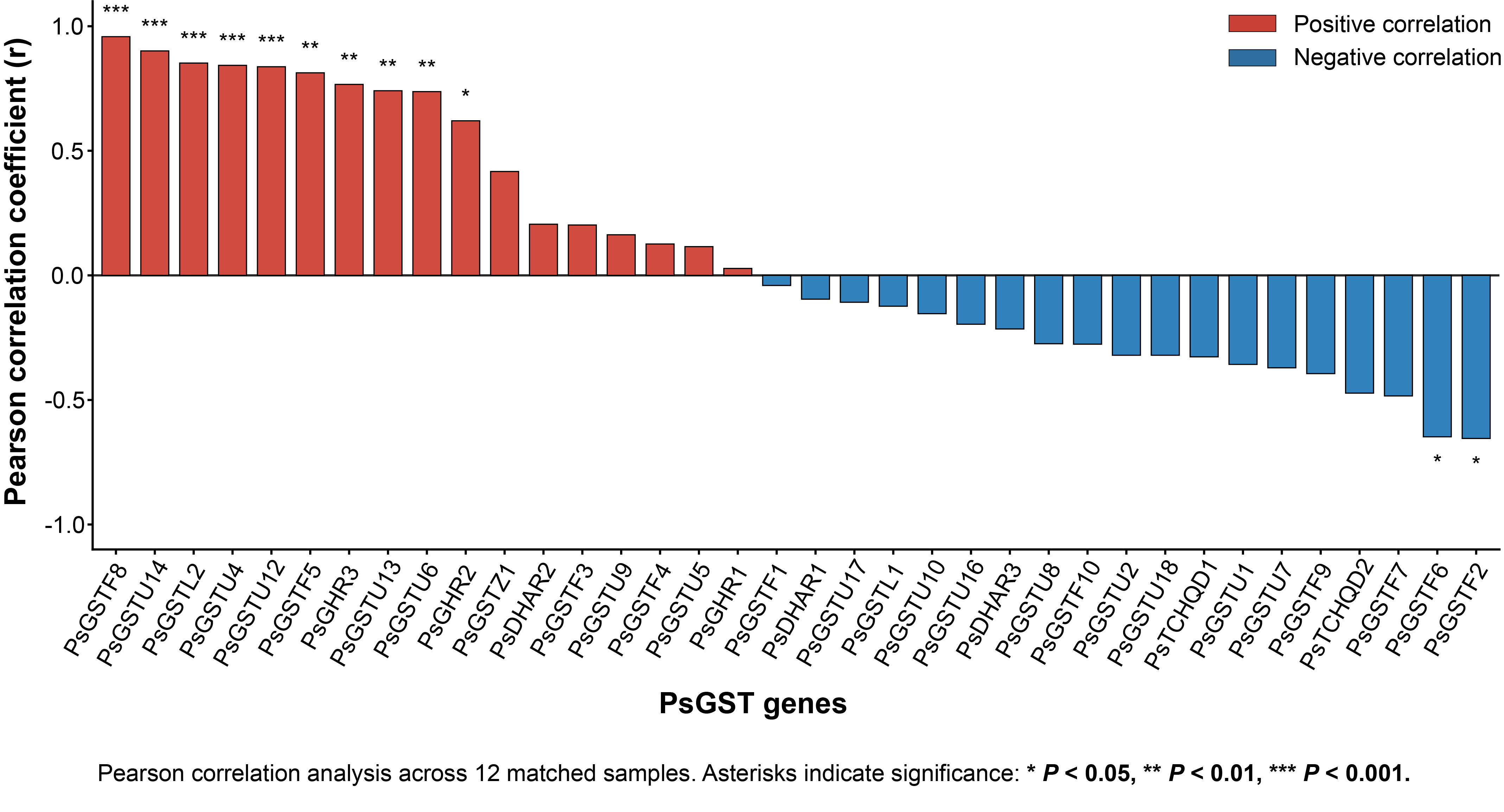

Supplement: Supplementary file 1 [file biology-15-01002-s001.zip › supplementary Figure S4.png]
